# Development of ultrafast capabilities for X-ray free-electron lasers at the linac coherent light source

**DOI:** 10.1098/rsta.2018.0386

**Published:** 2019-04-01

**Authors:** Ryan N. Coffee, James P. Cryan, Joseph Duris, Wolfram Helml, Siqi Li, Agostino Marinelli

**Affiliations:** 1 SLAC National Accelerator Laboratory, Linac Coherent Light Source, Menlo Park, CA 94025, USA; 2 SLAC National Accelerator Laboratory, Stanford Pulse Institute, Menlo Park, CA 94025, USA; 3 SLAC National Accelerator Laboratory, Menlo Park, CA 94025, USA; 4 Zentrum für Synchrotronstrahlung, Technische Universität Dortmund, Maria-Goeppert-Mayer-Straße 2, 44227 Dortmund, Germany; 5 Physik-Department E11, Technische Universität München, James-Franck-Straße 1, 85748 Garching, Germany; 6 Department of Physics, Stanford University, Stanford, CA 94305, USA

**Keywords:** X-ray free electron laser, attosecond, electron dynamics, charge migration, attosecondX-ray pulses

## Abstract

The ability to produce ultrashort, high-brightness X-ray pulses is revolutionizing the field of ultrafast X-ray spectroscopy. Free-electron laser (FEL) facilities are driving this revolution, but unique aspects of the FEL process make the required characterization and use of the pulses challenging. In this paper, we describe a number of developments in the generation of ultrashort X-ray FEL pulses, and the concomitant progress in the experimental capabilities necessary for their characterization and use at the Linac Coherent Light Source. This includes the development of sub-femtosecond hard and soft X-ray pulses, along with ultrafast characterization techniques for these pulses. We also describe improved techniques for optical cross-correlation as needed to address the persistent challenge of external optical laser synchronization with these ultrashort X-ray pulses.

This article is part of the theme issue ‘Measurement of ultrafast electronic and structural dynamics with X-rays’.

## 1. Introduction

During a chemical reaction, the motion of the atomic constituents is extremely fast, less than one trillionth of a second (10^−12^ s = 1 ps). In order to capture this ultrafast motion, researchers often employ a variant of stroboscopic imaging called ‘pump/probe spectroscopy’. In this variant, an ultrashort light pulse, typically less than 100 fs in duration (1 fs = 10^−15^ s), first initiates or ‘pumps’ the photo-chemical reaction, by exciting the system to a non-equilibrium state. A second ultrashort light pulse then probes the resulting dynamics. Instead of taking many exposures of a single reaction, as is typical in stroboscopic imaging, experiments are repeated many times, with a varying temporal delay between the pump and probe pulses, in order to map out the time-dependent motions during the photo-induced reaction.

Ultrafast pump/probe techniques are now routinely used with optical pulses to follow chemical reactions on the time scale intrinsic to dynamics of the atomic nuclei in the molecule. This has led to the development of the field of ‘femtochemistry’ [1]. This nuclear motion, however, is rather slow compared with far more rapid motion of the electrons, whose charge provides the screening to stabilize molecules, and whose redistribution initiates all chemical changes, including isomerization and dissociation. The natural timescale for electron motion in atoms and molecules is about 100 times faster than the timescale for nuclear motion: electrons move across a molecular bond in 0.1–1 fs [2–4]. Dynamics on this timescale is a science frontier both for experiments and for quantum many-body theory [5–7].

Ultrafast optical spectroscopy has revolutionized our understanding of the time-domain dynamics in atoms, molecules and solid-state systems. However, the development of ultrafast soft X-ray pulses is an important technological advance because soft X-ray pulses interact most strongly with core electrons, which are highly localized at the atomic centres in molecular systems. Addressing these core electrons is highly preferable, because their binding energy and core-to-valence absorption spectrum provide a sensitive measure of localized electron density around various atoms in a molecule or molecular complex [8–12]. Probing the local chemical environment with atomic-site specificity is highly advantageous for studying ultrafast charge dynamics, because the charge variations throughout the molecule tend to be localized at different atomic sites in the molecule [13]. With an eye towards rational chemical design, the emerging desire to follow electronic motion in chemically active molecular systems has motivated the strong push for soft X-ray spectroscopy on the timescale of such electron motion, e.g. from femtoseconds to attoseconds.

The ability to produce ultrashort, high-brightness X-ray pulses with free-electron lasers (FELs) has further revolutionized the field of ultrafast spectroscopy. From the first lasing of the Linac Coherent Light Source (LCLS), there have been significant efforts devoted towards improving the temporal resolution of X-ray pump/probe experiments. Starting from a time-resolution of approximately 120 fs available for the first time-resolved experiments at LCLS [14], the development of femtosecond bunch shaping, two-colour FELs and femtosecond time-stamping have improved our ability to resolve time-dependent processes down to the femtosecond scale. At the same time, ongoing experiments are aiming to extend pump/probe capabilities to the sub-femtosecond regime.

In this paper, we review the development of femtosecond capabilities at the LCLS and present the current effort to reach sub-femtosecond timescales. The paper is organized as follows: in §2 we discuss the temporal properties of X-ray FELs and present the experimental development of single-pulse femtosecond pulse shaping and two-colour X-ray pump/X-ray probe capabilities at LCLS. We also discuss the generation of single-spike attosecond hard-X-ray pulses and introduce the ongoing work towards the generation of pulses with sub-femtosecond duration at soft X-ray wavelengths. In §3, we discuss the development of femtosecond timing and synchronization techniques and present the ongoing effort to push experimental measurements below the femtosecond barrier using optical streaking techniques.

## 2. X-ray pulse production

### (a) Temporal properties of X-ray free-electron lasers

In an X-ray free-electron laser (XFEL), a relativistic electron bunch travels in a magnetic undulator and undergoes a collective instability which results in the exponential growth of radiation power, a process called FEL instability [15,16].

The central wavelength emitted by an FEL is given by the resonance condition:

2.1 $$\lambda_{r}=\frac{\lambda_{w}}{2\gamma^{2}}\left(1+\frac{K^{2}}{2}\right),$$

where *λ*_*r*_ is the radiation wavelength, *λ*_*w*_ is the undulator period, *K* is the undulator parameter (typically of order 1 for X-ray lasing) and *γ* is the electron beam's Lorentz factor. When the resonance condition is satisfied, the radiation field slips ahead of the electron bunch by one wavelength per undulator period.

The FEL gain length *L*_*g*_ is defined as the length over which the radiation power grows by a factor *e*. The slippage length accrued in a gain length is called the cooperation length *l*_*c*_ [17]:

2.2 $$l_{c}=\lambda_{r}\frac{L_{g}}{\lambda_{w}}.$$

In an FEL working on the principle of self-amplification of spontaneous emission (SASE), the FEL instability is triggered by noise and the temporal radiation distribution is composed of several spikes that are uncorrelated with respect to each other. Since the slippage process transports phase information across the electron bunch, the coherence length of a SASE FEL is given by the cooperation length. The shortest possible pulse that can be generated in a SASE FEL is a single-spike pulse, which means that the cooperation length is also the lower limit for the pulse duration of a SASE FEL [17].

In the one-dimensional FEL model, the cooperation length scales with the photon energy as

2.3 $$l_{c}\propto E_{\mathrm{ph}}^{-1/2}.$$

When including thermal effects and diffraction the exact dependence of the cooperation length is more complicated (e.g. [18,19]); however, it is true for most cases of practical interest that the cooperation length is a decreasing function of the photon energy. This implies that shorter pulses can be generated at hard X-rays than at soft X-rays. At the LCLS, the cooperation length is typically around 1 fs–2 fs for soft X-ray energies and few hundreds of attoseconds for hard X-rays [16].

### (b) Femtosecond shaping of electron bunches

The pulse duration in a SASE X-ray FEL is given by the longer of the two lengths discussed in the previous section: the cooperation length or the electron bunch length. State-of-the-art linear accelerators used to drive XFELs can easily generate bunches with a length of few tens of femtoseconds (e.g. [20]). To achieve a shorter pulse duration, one needs to either generate shorter electron bunches or to shape the bunches themselves so that only a small fraction of the available charge is used in the FEL.

Low-charge operation is a possible way to reduce the X-ray pulse duration. This can be understood as follows: the maximum electron beam peak current that can be used in an FEL is limited by collective effects such as coherent synchrotron radiation and the microbunching instability [21]. Typical X-ray machines operate in the range of 1–5 kA. Since there is a maximum achievable or usable peak current, it follows that the electron bunch duration can be reduced by operating the linac with lower charge per bunch. Low-charge operation has been demonstrated as an effective tool to generate X-ray pulses with approximately 10 fs duration [22,23].

Changing the electron beam charge is a time-consuming process that can take several hours of precious beam time. Selective spoiling of the electron bunch is a quicker and more effective method to shorten the X-ray pulse duration. Selective spoiling consists of decreasing the electron beam brightness in a time-dependent fashion so that only a short fraction of the electron bunch has the required beam quality for lasing. An emittance spoiler accomplishes this effect by scattering the particles through a thin metal foil [24,25], therefore increasing the transverse emittance well above the lasing tolerance. To effect this, a slotted aluminium foil is placed in the middle of a compression chicane, where the electron beam is both chirped and dispersed. The resulting electron beam distribution has a strong correlation between time and horizontal position, allowing for selective spoiling of the transverse emittance in the time domain.

To extend the concept of time-selective spoiling to high repetition rates the idea of optical shaping of XFELs was developed. In this case, the longitudinal emittance is selectively increased using a laser heater [26]. A laser heater is typically employed to control the energy spread of the electron bunch and suppress the microbunching instability [27]. Using a temporally shaped laser heater, one can increase the energy spread beyond the acceptable value for lasing in selected parts of the electron bunch, therefore shaping the X-ray profile and allowing for X-ray pulses considerably shorter than the electron bunch length. The optical shaping method naturally scales to high repetition rates (approx. 1 MHz), since no solid target is inserted into the beam path.

**Figure 1.**
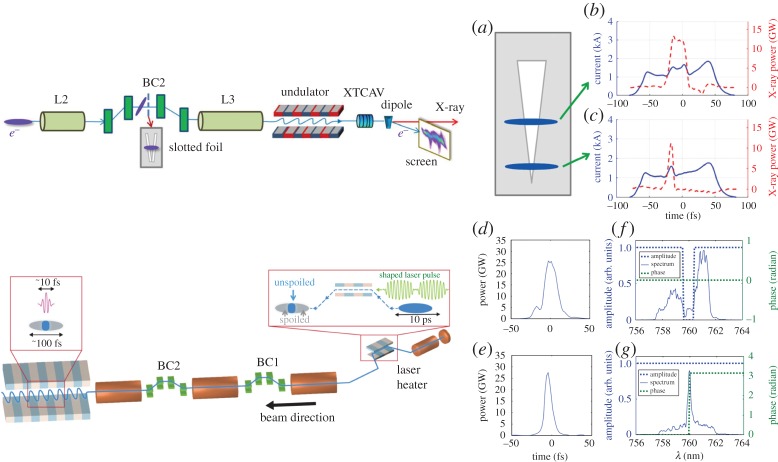
Schematic view (top, left side) of the emittance spoiler (*a*) and X-ray temporal profile (*b* and *c*) measured with a transverse cavity for different spoiler settings (from [25]). Schematic view (bottom, left side) of the optical shaping scheme and X-ray temporal profile (*d*,*e*) measured with a transverse cavity for different laser heater shapes, amplitude and phase (*f*,*g*) measured after amplification (from [28]). (Online version in colour.)

Figure 1 shows a schematic of both the emittance spoiler and the optical shaper, as well as the measured X-ray temporal profiles using both methods.

### (c) Attosecond pulses

**Figure 2.**
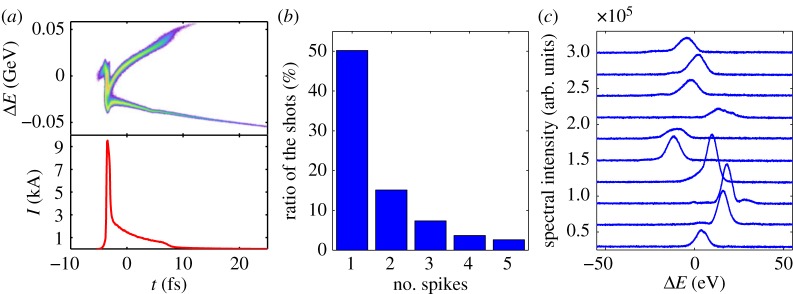
(*a*) Simulated longitudinal phase space (top) and current profile (bottom) of the nonlinearly compressed electron beam. (*b*) Percentage of shots with a given number of SASE spikes in the nonlinearly compressed electron beam. (*c*) Examples of measured single-spike spectra [30]. (Online version in colour.)

Owing to the natural scaling of the cooperation length, equation (2.3), sub-femtosecond pulses can be generated at hard X-ray energies simply by generating a short (<1 fs) electron bunch. Two techniques (detailed in table 1) have been shown to generate sub-femtosecond hard X-ray pulses. The first is the emittance spoiler described in the previous paragraph. With a simple modification of the beam optics in the bunch compressor, one can reduce the bunch duration to the single-spike FEL length, and therefore achieve sub-femtosecond pulses [29]. The second technique relies on the nonlinear compression of a low charge (20 pC) electron bunch [30]. Nonlinear compression is achieved by introducing a nonlinear chirp in the electron bunch prior to going through the bunch compressor system, which results in a short high-current spike at the head of the electron bunch. Both techniques result in single-spike multi-eV spectra, with an estimated pulse duration below 1 fs and a pulse energy of a few microjoules. Figure 2 shows data from the non-linear compression experiment. The spectra are dominated by single-spike events with a bandwidth of roughly 10 eV.

**Table 1.** Pulse properties for attosecond hard X-ray capabilities at 5.6 keV at the LCLS.

method	pulse energy	bandwidth	estimated pulse duration
emittance spoiler	10 μJ	10.5 eV	230 as
nonlinear compression	10 μJ	4.5 eV	420 as

In the soft X-ray range generating sub-femtosecond pulses requires an additional beam gymnastics to overcome the limitations due to slippage. Enhanced SASE was proposed by Zholents as a means to improve the gain length of X-ray free-electron lasers by compressing parts of an electron bunch with an infrared laser [31,32]. Figure 2 shows a schematic of this process: an electron bunch travels through a magnetic wiggler and interacts with a high power infrared pulse. The interaction results in an energy modulation which is subsequently converted into a density modulation, producing high-current spikes with a duration of 1 fs or shorter. The ESASE compression happens in a small chicane, typically using a dispersion of a factor 100 less than the bunch compressors in the linac. As a result, the collective effects that limit the beam quality in compressed electron bunches are negligible and one can achieve a shorter gain length. As a corollary, ESASE also results in a shorter cooperation length, therefore enabling the generation of sub-femtosecond pulses at soft X-ray energies. ESASE amplification can also be cascaded over several high-current spikes, allowing the generation of attosecond pulses with TW-scale peak power [33–35].

An alternative scheme called chirp/taper has been proposed by Saldin [36]. In a chirp/taper scheme, the electron beam is also modulated by a high-power infrared pulse. However, instead of compressing the bunch, Saldin proposed the use of a taper to maintain the resonant condition only on a short fraction of the electron bunch. To understand this method, consider an electron bunch with a linear energy chirp, i.e. with the following energy distribution:

2.4 $$\gamma (s)=\gamma_{0}+\frac{d\gamma_{b}}{ds}(s-s_{0}),$$

where *γ*(*s*) is the Lorenz factor as a function of the longitudinal coordinate *s*, *γ*_0_ is the mean beam Lorentz factor and *s*_0_ is the location of the centre of the bunch. As the radiation generated at some location *s*_1_ slips ahead of the electron bunch it finds electrons of different energy, causing a mismatch between the radiation wavelength and the local resonant wavelength. This wavelength mismatch can be corrected, if the undulator parameter is also changing so that, as the radiation slips ahead of the electrons, the resonance condition is maintained [36–38]:

2.5 $$d\lambda_{r}=\frac{\partial \lambda_{r}}{\partial K}\frac{dK}{dz}\lambda_{w}+\frac{\partial \lambda_{r}}{\partial \gamma }\frac{d\gamma_{b}}{ds}\lambda_{r}=0.$$

If the energy distribution is sinusoidal instead of linear, then the chirp/taper matching condition can only be satisfied locally, and lasing is restricted to one or more short fractions of the electron bunch resulting in one or more short X-ray pulses. The chirp/taper scheme does not necessarily improve the cooperation length, however the strong frequency chirp in the X-rays can be exploited to shorten the pulse duration using a spectral filter. Furthermore, the chirp/taper scheme can be driven into the post-saturation superradiant regime, considerably reducing the pulse duration with respect to the cooperation length limit [39].

While in the literature ESASE and chirp/taper were proposed as two independent methods, in practice, one cannot implement ESASE without using a chirp/taper scheme at the same time. This is due to the strong longitudinal space-charge forces generated by the ESASE spikes, which result in a correlated energy spread considerably larger than the FEL bandwidth [40]. It can be shown that the nonlinear terms in the energy distribution induced by space charge contribute to shortening the X-ray pulse. This effect can be exploited by allowing the current spike chirp to grow prior to the FEL instability, in a scheme called space-charge boosted ESASE.

An ESASE experiment is currently underway at the LCLS. The XLEAP project (X-ray Laser-Enhanced Attosecond Pulse generation) aims at generating sub-fs pulses in the energy range between 400 and 1000 eV. The experiment employs a variable-gap wiggler with a period of 32 cm and an undulator parameter up to *K* = 52. The energy modulation can either be induced by a 2 μm laser based on Ho:YLF amplifiers, or by the coherent undulator radiation emitted by the electron bunch [41]. A magnetic chicane with a longitudinal dispersion up to *R*_56_ = 1 mm is employed to generate high current spikes.

**Figure 3.**
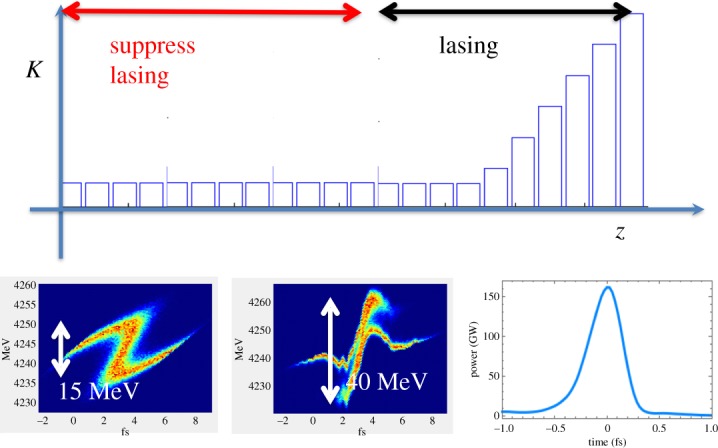
Schematic of the XLEAP sub-fs experiment. A short high-current spike is generated in the electron bunch by interaction of the beam with a high-power laser. The electron beam is kicked transversely to suppress lasing in all but the last eight undulator sections, allowing space charge to introduce a large energy chirp. Finally, the orbit is straightened and the electron beam is sent through a tapered undulator for the generation of sub-femtosecond pulses. (Online version in colour.)

**Figure 4.**
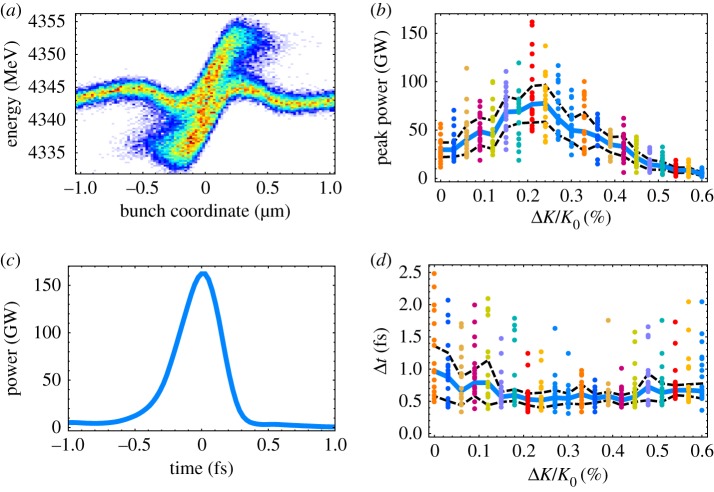
Numerical simulations of the XLEAP space-charge boosted ESASE scheme. (*a*) Longitudinal phase space of the compressed ESASE spike after a drift length of 40 m. (*b*) Peak power as a function of undulator taper. (*c*) A single-shot temporal profile. (*d*) Pulse duration as a function of undulator taper. (Online version in colour.)

Figure 3 shows a schematic of the FEL scheme. To maximize the available bandwidth and minimize the pulse duration, the longitudinal space-charge field is used to introduce a chirp on the current spike. To this end, the FEL instability is suppressed in the undulator with a large orbital kick. After a certain length, the beam orbit is straightened and the undulator is tapered to match the space-charge induced chirp and generate a sub-femtosecond FEL pulse. Figure 4 shows the results of numerical simulations of the XLEAP experiment at 800 eV, which suggest that pulses as short as 0.5 fs can be achieved with the space-charge boosted ESASE method.

We note that the longitudinal space-charge field in an undulator or a wiggler is proportional to 1 + *K*^2^/2. In future implementations of this scheme, a more elegant and efficient way to control the space-charge induced chirp would be the use of a dedicated wiggler magnet after the ESASE chicane and prior to the FEL undulator.

### (d) Two-colour X-ray pulses

In recent years, the use of two-colour X-ray pulses for ultrafast pump/probe experiments has been the subject of intense investigation at LCLS and other FEL facilities worldwide. A two-colour FEL can generate two X-ray pulses of different photon energy and with a variable delay. We recall that the resonant wavelength in an FEL is given by equation (2.1). Here, the undulator parameter *K* and the beam's Lorentz factor *γ* are both tunable quantities, while period *λ*_*w*_ is typically a fixed parameter. With this in mind, two-colour FELs can be divided into two categories: multi-bunch FELs, where different beam energies are used to make pulses of different colours; and multi-undulator schemes, where undulators with different magnetic fields are used.

#### (i) Multi-bunch X-ray FELs

**Figure 5.**
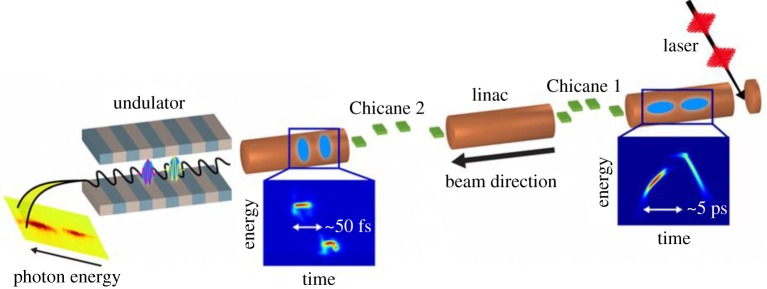
Multi-bunch X-ray free-electron lasers rely on the generation of two or more bunches by splitting the injector laser into multiple pulses (from [42]). (Online version in colour.)

Multi-bunch FELs rely on the generation of more than one electron bunch at the injector. Figure 5 shows a schematic of a typical multi-bunch scheme. Typically multiple bunches are generated by splitting the photocathode drive laser that generates the electron beam into more pulses, each extracting one electron bunch from the cathode.

Multi-bunch FELs can operate in two regimes: the single-bucket and the multi-bucket regime. In the first case, the two bunches are generated with an initial delay much smaller than the radio frequency wavelength of the accelerator. This method is called the twin-bunch FEL [42]. For the s-band LCLS accelerator, the accelerating period is 350 ps and the two bunches are initially delayed by several picoseconds. As the two bunches are accelerated and compressed in the LCLS linac, they acquire different energies according to the differing instantaneous amplitude of the accelerating radio frequency field and their time delay is reduced to few tens of femtoseconds. Typically, the energy separation is limited by the energy acceptance of the beam transport system to roughly 1%. The time delay can be controlled independently of the energy separation in the range of 0–100 fs, with an arrival time jitter of order 5 fs. In multi-bucket schemes, the electron bunches are delayed by multiples of the accelerating period. Different bunch energies are achievable by either injecting the bunches at different phases or by employing the finite width of the radio frequency accelerating pulse [43,44].

Since each colour is generated by an independent electron bunch, multi-bunch schemes can generate pulse energies comparable to the standard single-colour operation (of order 1 mJ at LCLS). Furthermore, each colour uses the entire electron bunch, making these schemes the favourite method for operation at high photon energies, where the saturation length is comparable to the available undulator length.

#### (ii) Multi-undulator schemes

**Figure 6.**
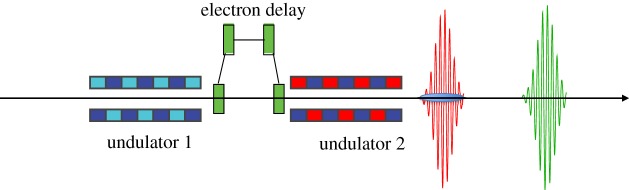
Typical multi-undulator scheme. The electron bunch travels through two undulators with different *K*, emitting X-rays of different colours. A magnetic chicane is used to delay the electron bunch, therefore delaying the arrival time of the second X-ray pulse. (Online version in colour.)

Multi-undulator schemes employ undulators with different *K* to generate pulses of different photon energy. The first two-colour X-ray scheme was the split undulator [45–47], shown in figure 6. The undulator is divided into two parts with different *K* and a magnetic chicane is used to delay the electron bunch, introducing a delay between the X-ray pulses emitted in each undulator. In this scheme, the energy separation is only limited by the tunability range of the undulators. The SACLA team has demonstrated two-colour generation with large energy separation employing variable gap undulators [47]. The maximum allowable time-delay depends on the strength of the magnetic chicane and can easily reach 1 ps with a compact design.

The main drawback of this method is that the same electrons are used for generating both colours, which means that saturation cannot be achieved on either colour, resulting in a loss of an order of magnitude in peak power. A way to improve this scheme is to allow only a fraction of the electron bunch to lase in each undulator, therefore allowing the FEL to reach saturation for both colours [48]. This idea is generally termed ‘fresh-slice lasing’. This can be achieved by introducing a time-dependent transverse kick and allowing only a slice of the electron bunch to travel on a straight orbit in each undulator. Time-dependent kicks can be launched either by capitalizing the transverse wake of a corrugated waveguide, or by chirping the electron bunch and introducing transverse dispersion in the beam transport [49,50]. Alternatively, the same effect can be obtained by employing time-dependent defocusing from a corrugated structure [51] or by seeding different slices of the electron bunch with different seed wavelengths and amplifying them separately in two undulators [52].

Fresh-slice schemes allow for maximum flexibility in tailoring the X-ray pulses while still allowing the FEL to reach the full saturation power. A temporal resolution of order 10 fs and pulse energies in the hundreds of microjoule can be achieved with fresh-slice FELs, while the time delay can be varied continuously up to a maximum of a few picoseconds depending on the exact design of the magnetic chicane.

## 3. Pump/probe timing: from femtoseconds to attoseconds

### (a) The femtosecond regime

Achieving few- to sub-femtosecond X-ray pulses is only part of what is required to make ultrafast measurements at the LCLS Free Electron Laser (FEL) facility. Synchronization between X-ray pulses produced by the FEL and optical pump or probe pulses from an external laser system continues to be a persistent challenge since the temporal jitter between the optical and X-ray pulses directly limits the overall time resolution of a pump–probe measurement. In traditional femtosecond and attosecond measurements, both the pump and the probe are derived from the same laser source. By contrast, FELs use conventional feedback techniques to stabilize the optical laser pulse arrival time relative to a radio frequency (RF) reference from the accelerator. Owing primarily to thermal effects, RF noise and energy jitter in the electron bunch, the electron bunches accumulate additional temporal jitter as they propagate along the acceleration and bunch compression chain. In particular, the energy jitter is directly transformed into a timing jitter in the magnetic chicane bunch compressors. The resulting total accumulated temporal jitter can be as extreme as 120 fs RMS at the LCLS [14] or as minimal as 20 fs RMS [53] for the higher repetition rate FELs.

#### (i) Spectral encoding of X-ray/optical delay

A number of techniques have been developed for measuring the X-ray/optical arrival time at the LCLS. These techniques rely on the measurement of transient optical properties (index of refraction) of a material exposed to X-ray pulses, and broadly fall into two categories dubbed spatial or spectral encoding. In the case of the spatial encoding technique [54–56], a short optical pulse is crossed with the X-ray pulses in an interaction medium and the change in the material reflectivity or transmission is measured. The convolution of the X-ray pulse duration and the temporal duration of the optical pulse both contribute to the measured signal width, and thus the overall resolution of the arrival time measurement. For these techniques, the measurement window, which limits the temporal range of the measurement, is fixed by the spatial diameter of beam overlap. By contrast, spectral encoding techniques imprint the change in transmission on a highly chirped super-continuum pulse. Thus, a measure of the delay comes from the chirp-controlled monotonic wavelength to time mapping [57]. One benefit of this spectral mapping is the adjustable temporal range as well as the independence of spatial beam profile for either X-rays or optical beams.

When moving to a solution for the upcoming high repetition rate FEL sources like LCLS-II, the interaction material becomes a primary concern. X-ray fluence levels of order 50–100 mJ cm^−2^ at the sample are typical of 10% Δ*R*/*R* [55] in traditional spatial reflectivitity and spectral transmission measurements. This is a level that is uncomfortably close to the materials damage threshold and in fact the interaction materials, typically YAG (yttrium aluminum garnet, Y_3_Al_5_O_1_2) or Si_3_N_4_, already show discoloration and damage during experimental exposure at 120 Hz operation. Clearly, a much more X-ray dose tolerant material with sufficient thermal transport is needed as we move to the high average power of the LCLS-II.

**Figure 7.**
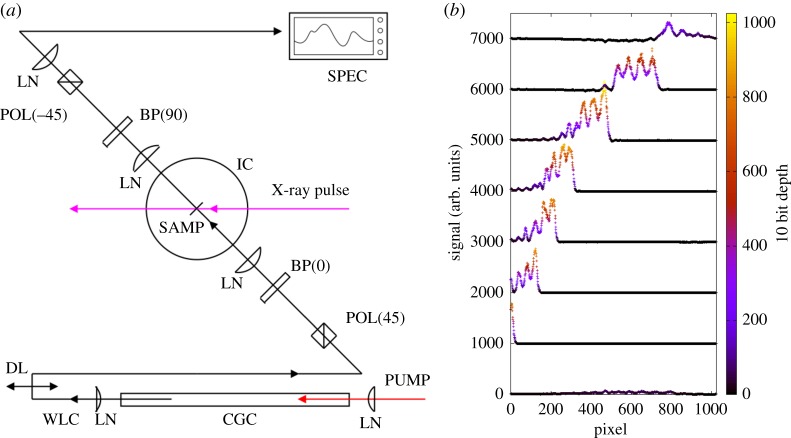
(*a*) Schematic of the interference mode of spectral encoding. Here birefringent plates BP(*h*/*v*) are used to delay and then re-time crossed polarization components of white light continuum (WLC) from a continuum generation cell (CGC). The residual elliptical polarization due to the X-ray modulation of the refractive index then passes through the otherwise crossed polarizer (POL(−45)) and is then analysed in a spectrum analyser. Owing to the spectrum to time mapping, [57], the wavelength that is passed through the crossed polarizer is monotonically mapped to time. (*b*) Example ‘waterfall’ plot of preliminary results of the interference mode of spectral encoding at the LCLS in 5 μm thick diamond sample with 1800 eV photon energy. (Online version in colour.)

It has been recently demonstrated that an interference mode of spectral encoding (figure 7) allows one to measure the induced refractive index change in diamond at X-ray fluence levels that are two orders of magnitude lower than that required for the traditional measurements. Not only does this allow for less energy deposition in the material, it also allows for the use of a thin diamond transmission window as an interaction medium, which has a large bulk thermal conductivity [58,59]. The interference scheme uses crossed and matched birefringent plates to temporally shear an originally 45° linear polarization into two crossed polarization states (*h* and *v*) before the interaction material. As a result, the X-ray induced refractive index changes causes a step change in the spectral phase for wavelengths that arrive at the crystal after the X-ray pulse. Owing to the birefringent shear, there is a region of spectrum where one polarization state is transmitted through the material ahead of the X-ray pulse while the other polarization state trails the X-ray pulse. Upon subsequent birefringent compensation of the original shear, nearly all spectral regions recombine to the original 45° polarization. Only the portion of the spectrum where one polarization leads and the other lags the X-ray pulse will show an elliptical result that gives power through an analyser set to −45°.

Pipelined high throughput of a spectral encoded timing signal could allow for individual timing measurements to be made available for on-the-fly processing. One could thereby imagine novel time-domain methods of analysing data such as in ref. [60] that could occur inside the detector buffer memory before data transfer. This would potentially allow very heavy compression of area detector signals. Under such a mode of operation, rather than stacking images into on-board memory, the timing measurement would allow each image to be sorted into an appropriately ordered time bin. Then at a much lower rate, when enough sorted frames have accumulated in the buffered histogram, that histogram could be passed through the Fast Fourier Transform (FFT) such that only the relevant Fourier coefficients need to be pushed into the data stream. This paradigm, however, requires that the latency of the timing determination be made comparable to the repetition rate in the system, and thus the sub-10 μs target range.

Another, simpler paradigm, is that the principle detector will again fill a histogram with one such bin associated with optical/X-ray delay. In this scheme, one can imagine retaining a running sum of frames that have been accumulated into each of the bins. As some bins fill faster than others the principle detector could preferentially veto new frames associated with high statistics bins. In this way, one could use the bias of even statistical representation in the delay bins to intelligently veto over-represented delay configurations. As before, in this mode one would prefer not simply a high throughput, but rather a very low latency in conversion from jitter measurement to analysed optical/X-ray delay.

Given the desires for low latency on the microsecond scale, an integrative approach to the measurement, sensor and analysis is highly desirable. Sensor considerations along with data handling on the sensor chip drive us to find a low bit-depth solution to the measurement itself. Herein lies another benefit of an interference based scheme for the arrival time measurement. Figure 7*b* shows preliminary results of the spectrally encoded readout of the modulation signal. The ripples on the signal arise from the optical etalon effect of the 5 μm thick diamond sample. Owing to the traditional spectrum-to-time mapping, the spectral location of the region of incomplete extinction, here measured in pixels of the spectrometer sensor, provides the measurement of delay. This is seen from the increasing delay between X-ray and optical pulses shown as a ‘waterfall’ plot. The appearance of this signal against a dark background allows us to use a low bit-depth sensor, in stark contrast with the non-interferrometric case that required well-behaved background subtraction in order to uncover the typical few percentage of signals. The new interference scheme is not expected to significantly improve the temporal resolution of spectral encoding (approx. 5–10 fs RMS currently [57,61]), but rather to extend its dynamic range to significantly weaker pulses, thus accommodating the much lower pulse energy but much higher average power of the LCLS-II. Furthermore, the signal visible in figure 7*b* was derived from 1800 eV soft X-ray photon absorption in CVD diamond, a sample that shows no measurable signal in the traditional method. For LCLS-II, deriving a timing signal from a reasonably X-ray transparent material with very high thermal conductivity will allow the continuous arrival time diagnostic even in the high average power beams. An area under active development is the extension of the spectrogram encoding method of [62]. Recent progress leveraging analysis of streaming image data from high frame-rate sensors is likely to give promising results for such a method of recovering the finest time resolution, even sub-femtosecond, between optical and X-ray pulses.

#### (ii) X-ray pulse duration diagnostics

As the timescales become shorter for pump/probe measurements, crossing into the attosecond regime, experiments require a more precise knowledge not only of the relative delay between the pump and probe laser pulses, but also the temporal profiles of the X-ray laser pulses themselves. Owing to the stochastic nature of the SASE process, the temporal structure of XFEL pulses is typically chaotic and each X-ray pulse is inherently different from adjacent shots. This necessitates a single-shot measurement technique for determination of the temporal structure of SASE X-ray pulses. Moreover, it is highly preferable if these measurements can be made in a non-destructive way, so that the temporal profile of the X-ray pulse can be diagnosed at the same time as pump/probe measurements are performed.

**Figure 8.**
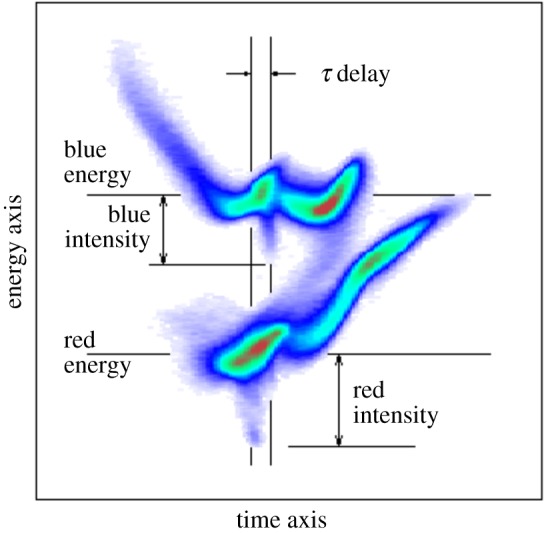
Annotated example image of an XTCAV measurement, used to diagnose the twin-bunch method of producing two-colour XFEL beams. (Online version in colour.)

A somewhat indirect route to diagnosing the temporal structure of the X-ray pulse is by measuring the FEL-induced lasing effects on the electron beam. To make such a measurement at LCLS, an X-band radio frequency transverse deflector along with an electron beam energy analyser (XTCAV) have been installed in the electron dump to measure the electron beam longitudinal (time-energy) phase space [23]. The FEL lasing process induces energy losses in the electron bunch, which are easily seen in the analyser. By mapping the energy loss as a function of time, one can measure the temporal profile of the X-ray power. Figure 8 shows an example of the longitudinal phase space of the electron beam recorded in the XTCAV apparatus. Though this is an indirect measure of the X-ray pulse parameters, the imprint of the lasing process on the spent electron bunch can be correlated with experimental results as well as machine parameters to inform veto or sorting algorithms [63]. Nevertheless, with the megahertz repetition rate operation of LCLS-II in mind, this method is severely limited by the read-out electronics of the camera in the XTCAV analyser, currently working at a 120 Hz frame rate. There are a number of other limitations to the XTCAV device. Currently, the resolution of the device installed at the LCLS is of the order of 1 fs RMS. Furthermore, there is a fundamental limitation to the resolution of this method given by slippage. The energy loss of the electron bunch mimics the temporal profile of the X-rays only if one is concerned with time scales that are longer than the cooperation length (equation (2.3)) or the slippage length after saturation (whichever is longer). X-ray emission is not an instantaneous effect and slippage can lengthen the pulse duration with respect to the temporal structure of the electrons. Therefore, the XTCAV could not be used for diagnosing single-spike sub-fs pulses, even if the longitudinal phase-space of the electrons could be measured with enough temporal resolution.

### (b) Optical streaking

Another possible route to a single-shot diagnostic of the X-ray beam temporal profile is through laser dressed photoelectron spectroscopy, or streaking, which provides a more direct measurement of the X-ray pulse temporal profile. Briefly, the streaking technique relies on X-ray photoionization in the presence of a long-wavelength dressing laser field. An X-ray pulse with sufficiently high photon energy will ionize an atomic system, releasing photoelectrons with a kinetic energy equal to the difference between the photon energy and the ionization potential. This ionized electron will then be accelerated by the longer-wavelength dressing laser, and its momentum will be altered by this field. Variations in the measured momentum distribution will reflect the magnitude and direction of the dressing laser vector potential at the time of ionization. In this way, information about the temporal profile of the ionizing pulse will be encoded in the momentum distribution of the emitted photoelectrons. More specifically, the streaked electron momentum distribution contains real-time information on the time-energy structure of the outgoing electron wavepacket (EWP), including the subsequent processes which affect this EWP on ultrafast timescales [6].

#### (i) Linear streaking measurements

Initial experiments performed at FEL facilities employed a linearly polarized streaking laser to produce modulations in the measured photoelectron energy distribution [64–69]. For more information on the development of ultrafast pulse characterization at X-ray FEL facilities, see the recent review paper by Helml *et al.* [70]. Optical streaking experiments were not originally developed with XFEL facilities in mind. In fact, many of the techniques now implemented at XFELs were pioneered with high harmonic generation-based, table-top attosecond laser sources. The delay-dependent photoelectron spectrum (or spectrogram) of the photoionized electron wavepacket recorded in a two-colour ionization set-up can be used to reconstruct both the streaking laser temporal profile and the average X-ray pulse temporal profile, with a number of different algorithms [71]. However, experiments at XFEL facilities suffer many hardships that are not often encountered in table-top attosecond pulse generation. As mentioned earlier, XFEL pulses typically lack shot-to-shot reproducibility, and the timing synchronization is on the order of a few hundred femtoseconds. This necessitates a single-shot measurement technique, which can determine both the X-ray pulse temporal profile and the relative optical/XFEL pulse arrival time.

#### (ii) Attosecond angular streaking

In contrast to the linearly polarized laser field discussed above, which has a time varying amplitude with a constant direction, a circularly polarized laser field has nearly constant amplitude with a time-varying direction. This interesting property has led to the development of angular streaking (also called the ‘attoclock’ technique), which replaces the linearly polarized streaking field with a circularly polarized one, and encodes the temporal profile of the ionized electron wavepacket into the angular distribution of the photoelectron momenta [72–77]. The use of a circularly polarized dressing laser fields for attosecond pulse metrology was proposed by Zhao *et al.* [73], who considered the technique particularly useful for pulses produced via the polarization gating technique [71]. A similar technique was pioneered by Eckle *et al.*, who realized that angular streaking can be applied in a single-pulse modality to study strong-field ionization dynamics on the attosecond timescale [74].

**Figure 9.**
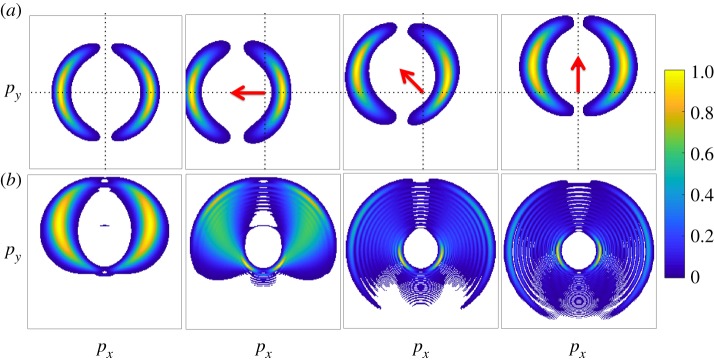
Simulated photoelectron momentum distributions resulting from two-colour ionization. This figure shows two-dimensional slices of the three-dimensional momentum distribution in the plane perpendicular to the X-ray propagation direction. (*a*) The results for a Fourier transform-limited Gaussian X-ray pulse with 300 as full-width-at-half-maximum duration. The angular distribution of the photoelectrons is a dipole pattern, characteristic of photoionization of a 1 s atomic orbital with linearly polarized X-ray pulses. In the left panel, the streaking field intensity is set to zero. Subsequent panels in the top row demonstrate the effect of the streaking laser field on the projected photoelectron momentum distribution for different delays between the X-ray pulse and the streaking laser field. The arrow indicates the direction of the instantaneous dressing laser vector potential at the peak of the attosecond pulse. (*b*) The effect of the X-ray pulse duration on the slice momentum distribution when the streaking laser field is directed along the *p*_*y*_ axis (as in the right panel of (*a*)). From left to right along (*b*), the X-ray pulse duration is 600 as, 1.2 fs, 2.4 fs and 4.8 fs. The simulation considers 25 eV photoelectrons interacting with a 1.3 μm laser field with *U*_*p*_ = 4 eV (where *U*_*p*_ = |*A*_0_|^2^/4 is the ponderomotive potential of the streaking laser field). The photoelectron momentum distribution intensity is normalized to 1. (Online version in colour.)

**Figure 10.**
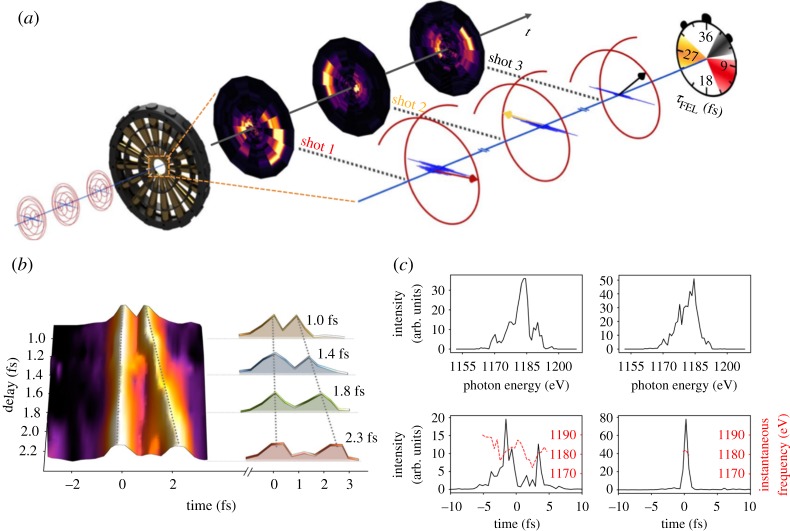
Figure adapted from ref. [75]. (*a*) Schematic of attosecond angular streaking at LCLS. (*b*) Double attosecond X-ray pulses sorted by delay. (*c*) Two representative pulse retrieval results, including X-ray spectrum and instantaneous frequency. (Online version in colour.)

**Figure 11.**
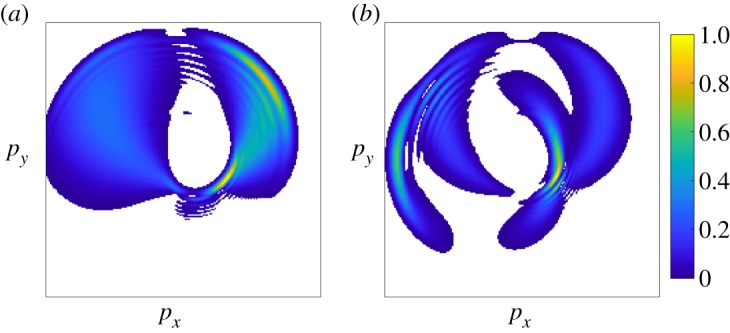
Photoelectron momentum distributions for more complicated X-ray pulse structures. (*a*) Simulation of a 300 as FWHM X-ray pulse chirped to 1.2 fs FWHM duration with the same streaking laser field as in figure 9. (*b*) Simulation of the streaked photoelectron momentum distribution from two 600 as FWHM X-ray pulses separated by a quarter of the streaking laser period. As in figure 9, the photoelectron momentum distribution intensity is normalized to 1. (Online version in colour.)

To better demonstrate the power of angular streaking for attosecond pulse metrology, figure 9*a* shows the calculated variation of the photoelectron momentum distribution as the delay between an ionizing X-ray pulse and a circularly polarized streaking pulse is varied. In this example, the X-ray pulse duration (300 as) is much shorter than the streaking laser period (approx. 4.3 fs at 1.3 μm wavelength), and it is clear to see that the photoelectron momentum distribution is offset in the direction of the instantaneous vector potential of the streaking laser field. As the X-ray pulse becomes longer (figure 9*b*), the angular spread of the photoelectron momentum distribution becomes larger. Eventually fringes begin to form, these patterns originating from the quantum interferences between photoelectrons released at different times, with different initial momenta, which are streaked by the vector potential to the same final momentum [76], similar to what has been observed in strong-field driven electron rescattering [78]. Variations of the spectral phase of the ionizing X-ray pulse (figure 11) will also lead to variations in the measured photoelectron momentum distribution. Such variations have been used to determine the time-energy information of ultrashort X-ray pulses generated by the LCLS. Figure 10*a* shows a schematic of the experimental set-up used in [75] to characterize the SASE operation of the LCLS FEL in low bunch charge mode [22,23]. This experiment used an angular array of 16 time-of-flight electron detectors to measure the two-colour photoelectron momentum distribution. Using this technique, individual pulses were characterized with attosecond temporal resolution, including double pulse delays (figure 10*b*) and pulse reconstruction with instantaneous frequency (figure 10*c*). Other detection geometries have been considered, for instance, the original ‘attoclock’ work by the Keller group used a coincidence detection geometry [74], while Li *et al.* have demonstrated a velocity map imaging spectrometer that could be used to image the photoelectron momentum distribution [79].

For X-ray pulses with simple temporal structure, for example a Gaussian temporal profile with only linear chirp, the X-ray pulse profile can be directly inferred from the measured momentum distribution, as suggested in [77]. As the temporal structure of the incident X-ray pulse increases in complexity, the patterns become much more difficult to interpret directly. Consider the simulation shown in figure 11, which shows photoelectron spectrum of a heavily chirped X-ray and a double pulse. In this case, a reconstruction algorithm must be employed. The method used in [75] is based on the forward transformation of a set of basis waveforms. This method supposes that the original X-ray pulse, *E*_*X*_(*t*), can be decomposed into a set of basis functions, *α*_*n*_(*t*), each with a complex coefficient, *c*_*n*_:

3.1 $$E_{X}(t)=\sum_{n}c_{n}\alpha_{n}(t).$$

Each basis function can be forward transformed (or streaked) to give a photoelectron momentum amplitude contribution, *f*_*n*_(***p***). The linearity of the streaking interaction with respect to the X-ray field implies that the experimental photoelectron momentum amplitude, *b*(***p***), is then a linear combination of the streaked basis functions [80,81]

3.2 $$b(p)=\sum_{n}c_{n}f_{n}\left(p\right),$$

where *c*_*n*_ is the same coefficient as in equation (3.1). The recorded photoelectron momentum distribution is then given by the square of this amplitude:

3.3 $$B(p)={\left|b\left(p\right)\right|}^{2}={\left|\sum_{n}c_{n}f_{n}\left(p\right)\right|}^{2}=\sum_{n}\sum_{m}c_{n}^{*}c_{m}F_{nm}(p),$$

where *F*_*n*,*m*_ = f*_n_*f*_*m*_. Using this ansatz, the problem of reconstructing the incident X-ray pulse reduces to finding the proper coefficients, *c*_*n*_, to minimize the difference between the retrieved and measured photoelectron momentum distribution. Owing to sub-optimal resolution in both the energy and angular data in the initial demonstration experiment, the original algorithm used by Hartmann *et al.* ignored many of the interference channels that can occur, and used an approximate expression,

3.4 $$B(p)∼\sum_{n}{\left|c_{n}\right|}^{2}F_{nn}(p).$$

The above equation then defines a linear system of equations, which can be solved with a variety of methods to determine the basis set coefficients, *c*_*n*_, and thus reconstruct the incident X-ray pulse via equation (3.1).

Whether solving equation (3.3) or (3.4), the choice of basis functions, *α*_*n*_(*t*), is flexible. However, a fitting algorithm will perform most reliably for a basis set where the basis set expansion (equation (3.1)) exhibits the most sparsity, i.e. the X-ray pulse can be represented with very few basis functions. In a subsequent work [81], it was suggested that the ideal basis for representing the wavepackets created by short-pulse ionization is the von-Neumann basis [82]. Using the von-Neumann basis, it is possible to solve equation (3.3). The use of increasingly sparse representation bases will ultimately allow for lean predictive inference engines that one hopes will bypass iterative reconstruction algorithms, using a machine learning methodology, in order to also provide X-ray pulse reconstruction for on-the-fly veto and sort decisions.

### (c) Site-specific window on photoionization and electron correlation dynamics

**Figure 12.**
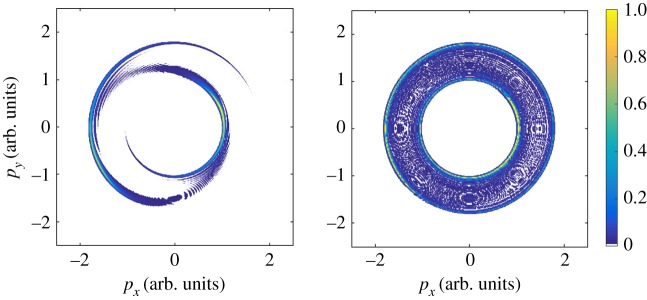
Simulated Auger electron momentum distribution resulting from core-level ionization with a 300 as X-ray pulse in the presence of a 2.4 μm (*T*_*L*_ = 8 fs), circularly polarized streaking field. The simulation is done in the strong-field approximation with a streaking field with *U*_*p*_ = 2.5 eV, and assumes an isotropic distribution of Auger electron emission. (*a*) An Auger lifetime of 1 fs, so that, *τ*_*X*_ < *τ*_*A*_ < *T*_*L*_. In (*b*), the Auger lifetime is 4 fs and comparable to the streaking laser period, i.e. *τ*_*X*_ < *τ*_*A*_∼*T*_*L*_. As in figure 9, the Auger electron momentum distribution intensity is normalized to 1. (Online version in colour.)

Beyond X-ray pulse characterization, angular streaking (and streaking experiments in general) can be used to probe photoionization dynamics on the attosecond timescale [4,6]. In one of the first experiments of attosecond physics, linear streaking was used to time-resolve the Auger process in krypton atoms [83]. The streaking method was also applied to study Auger cascades and resonant Auger decay in Kr [84,85]. Similar to the problem of X-ray pulse reconstruction, a circularly polarized streaking field is highly advantageous for time-resolving the Auger process. We demonstrate this utility in figure 12, which shows the calculated electron momentum distribution for an Auger electron with an initial kinetic energy of 1 a.u. (atomic unit), streaked by a 2.4 μm laser field. The lifetime of the intermediate core-excited state is varied between 1 fs, in figure 12*a*, and 4 fs in panel (*b*). In both panels, the excitation pulse is much shorter than the Auger lifetime, but the 4 fs Auger lifetime is approaching the dressing laser period (*T*_*L*_ = 8 fs). The simulations in figure 12 are performed in the strong-field approximation (SFA) using the same prescription as in the original work by Drescher *et al.* [83]. Recently, Kazansky *et al.* used a similar model to investigate the dependence of the measured electron momentum distribution on various parameters used in an experiment (the duration of the XUV pulse, the Auger decay time-of-life and the period of the dressing field) [86].

The streaked Auger electron momentum distributions are much more complex than what is observed in the case of an isolated attosecond pulse, and very strong interference patterns begin to emerge, even for short Auger lifetimes, as shown in figure 12*a*. Similar to the photoelectron streaking examples above, these features arise from interfering pathways, where electrons emitted with different momenta at different times end up with the same final momentum after interacting with the streaking laser field. The fringes are spaced by roughly the streaking laser photon energy [86], and so in order to emphasize these features the field-free energy of the Auger electron was set quite low, approximately 1 a.u. As the Auger lifetime approaches the dressing laser period, side-bands begin to form and the photoelectron momentum distribution becomes much more isotropic.

In order to retrieve temporal information on the Auger process, which is encoded in the two-colour photoelectron spectrum, it should be possible to apply the algorithm described in the previous section to reconstruct the Auger electron wavefunction with minimal modification. This type of experimental set-up would then add another dimension to time-resolved Auger spectroscopy [87], so that instead of simply studying the variation of Auger electron spectra as a function of a pump/probe delay, one could also study changes to the temporal profile of the Auger decay.

## 4. Conclusion

This work describes the most recent developments in the production and characterization of ultrashort X-ray pulses at the Linac Coherent Light Source. On the accelerator side most of the work has involved the temporal shaping of the lasing medium, i.e. the electron bunch. Control of the pulse duration down to the cooperation length limit has been achieved using emittance spoiling techniques. More flexible methods were demonstrated to simultaneously control the spectral and temporal properties with two-colour FELs, allowing X-ray pump/X-ray probe experiments with a resolution of order 10 fs. An effort to push beyond the cooperation length limit and enable attosecond pulses at soft X-ray energies is currently ongoing within the XLEAP project.

On the diagnostics side, we presented a few routes to high repetition rate determination of the temporal characteristics of the source. By tailoring our delay diagnostic of interference spectral encoding to low bit-depth measurements in high thermal conducting diamond samples, we enable autonomous decision-making for active sorting and intelligent veto of experimental results even before those results are ultimately transferred to permanent data storage. The same is true of the attosecond resolving X-ray pulse time-energy distributions; the full information about a given FEL pulse could thus be made available to automatic experimental control and guidance systems. Pushing the limits of temporal resolution and signal enhancement is sure to become increasingly important as we move to attosecond duration pulses at megahertz repetition rates. These new tools will enable the direct probing of electronic motions in molecular systems on their natural timescale.

## Data accessibility

This article has no additional data.

## References

[1] Zewail AH 1988 Laser femtochemistry. Science 242, 1645–1653. (10.1126/science.242.4886.1645) 17730575

[2] Leone SR *et al.* 2014 What will it take to observe processes in ‘real time’? Nat. Photonics 8, 162–166. (10.1038/nphoton.2014.48)

[3] Lépine F, Ivanov MY, Vrakking MJJ 2014 Attosecond molecular dynamics: fact or fiction? Nat. Photonics 8, 195–204. (10.1038/nphoton.2014.25)

[4] Vrakking M 2014 Attosecond and XUV physics: ultrafast dynamics and spectroscopy. In *Attosecond and XUV physics* (eds T Schultz, M Vrakking), pp. 1–16. New York, NY: John Wiley & Sons.

[5] Corkum PB, Krausz F 2007 Attosecond science. Nat. Phys. 3, 381–387. (10.1038/nphys620)

[6] Pazourek R, Nagele S, Burgdörfer J 2015 Attosecond chronoscopy of photoemission. Rev. Mod. Phys. 87, 765–802. (10.1103/RevModPhys.87.765)

[7] Kuleff AI, Cederbaum LS 2014 Ultrafast correlation-driven electron dynamics. J. Phys. B: At. Mol. Opt. Phys. 47, 124002 (10.1088/0953-4075/47/12/124002)

[8] Fahlman A, Nordling C, Siegbahn K 1967 ESCA: atomic, molecular and solid state structure studied by means of electron spectroscopy. Uppsala, Sweden: Almqvist and Wiksell.

[9] Stöhr J 1992 NEXAFS spectroscopy. Springer Series in Surface Sciences Berlin, Germany: Springer.

[10] Siegbahn K 1982 Electron spectroscopy for atoms, molecules, and condensed matter. Rev. Mod. Phys. 54, 709–728. (10.1103/RevModPhys.54.709) 17770242

[11] Catalin M, Paul M 2011 High-resolution inner-shell photoionization, photoelectron and coincidence spectroscopy. In *Handbook of High-resolution Spectroscopy* (eds M Quack and F Merkt). Hoboken, NJ: John Wiley & Sons (10.1002/9780470749593.hrs066)

[12] Wolf TJA *et al.* 2017 Probing ultrafast *ππ**/*nπ** internal conversion in organic chromophores via K-edge resonant absorption. Nat. Commun. 8, 29 (10.1038/s41467-017-00069-7) 28642477 PMC5481431

[13] Kuleff AI, Kryzhevoi NV, Pernpointner M, Cederbaum LS 2016 Core ionization initiates subfemtosecond charge migration in the valence shell of molecules. Phys. Rev. Lett. 117, 093002 (10.1103/PhysRevLett.117.093002) 27610850

[14] Glownia JM *et al.* 2010 Time-resolved pump-probe experiments at the LCLS. Opt. Express 18, 17 620–17 630. (10.1364/OE.18.017620) 20721148

[15] Bonifacio R, Pellegrini C, Narducci LM 1984 Collective instabilities and high-gain regime in a free-electron laser. Opt. Commun. 50, 373–378. (10.1016/0030-4018(84)90105-6)

[16] Pellegrini C, Marinelli A, Reiche S 2016 The physics of X-ray free-electron lasers. Rev. Mod. Phys. 88, 015006 (10.1103/RevModPhys.88.015006)

[17] Bonifacio R, De Salvo L, Pierini P, Piovella N, Pellegrini C 1994 Spectrum, temporal structure, and fluctuations in a high-gain free-electron laser starting from noise. Phys. Rev. Lett. 73, 70–73. (10.1103/PhysRevLett.73.70) 10056722

[18] Saldin EL, Schneidmiller EA, Yurkov MV 1993 On a linear theory of an FEL amplifier with an axisymmetric electron beam. Opt. Commun. 97, 272–290. (10.1016/0030-4018(93)90152-U)

[19] Xie M 2000 Exact and variational solutions of 3d eigenmodes in high gain FELs. Nucl Instrum. Methods Phys. Res. A 445, 59–66. (10.1016/S0168-9002(00)00114-5)

[20] Emma P *et al.* 2010 First lasing and operation of an angstrom-wavelength free-electron laser. Nat. Photon. 4, 641–647. (10.1038/nphoton.2010.176)

[21] Huang Z, Borland M, Emma P, Wu J, Limborg C, Stupakov G, Welch J 2004 Suppression of microbunching instability in the linac coherent light source. Phys. Rev. Spec. Top.-Accel. Beams 7, 074401 (10.1103/PhysRevSTAB.7.074401)

[22] Ding Y *et al.* 2009 Measurements and simulations of ultralow emittance and ultrashort electron beams in the linac coherent light source. Phys. Rev. Lett. 102, 254801 (10.1103/PhysRevLett.102.254801) 19659082

[23] Behrens C *et al.* 2014 Few-femtosecond time-resolved measurements of X-ray free-electron lasers. Nat. Commun. 5, 3762 (10.1038/ncomms4762) 24781868

[24] Emma P, Bane K, Cornacchia M, Huang Z, Schlarb H, Stupakov G, Walz D 2004 Femtosecond and subfemtosecond X-ray pulses from a self-amplified spontaneous-emission-based free-electron laser. Phys. Rev. Lett. 92, 074801 (10.1103/PhysRevLett.92.074801) 14995861

[25] Ding Y *et al.* 2015 Generating femtosecond X-ray pulses using an emittance-spoiling foil in free-electron lasers. Appl. Phys. Lett. 107, 191104 (10.1063/1.4935429)

[26] Marinelli A *et al.* 2016 Optical shaping of X-ray free-electron lasers. Phys. Rev. Lett. 116, 254801 (10.1103/PhysRevLett.116.254801) 27391728

[27] Huang Z *et al.* 2010 Measurements of the linac coherent light source laser heater and its impact on the X-ray free-electron laser performance. Phys. Rev. Spec. Top.-Accel. Beams 13, 020703 (10.1103/PhysRevSTAB.13.020703)

[28] Marinelli A *et al.* 2016 Optical shaping of X-ray free-electron lasers. Phys. Rev. Lett. 116, 254801 (10.1103/PhysRevLett.116.254801) 27391728

[29] Marinelli A, MacArthur J, Emma P, Guetg M, Field C, Kharakh D, Lutman AA, Ding Y, Huang Z 2017 Experimental demonstration of a single-spike hard-X-ray free-electron laser starting from noise. Appl. Phys. Lett. 111, 151101 (10.1063/1.4990716)

[30] Huang S *et al.* 2017 Generating single-spike hard X-ray pulses with nonlinear bunch compression in free-electron lasers. Phys. Rev. Lett. 119, 154801 (10.1103/PhysRevLett.119.154801) 29077438

[31] Zholents AA, Fawley WM 2004 Proposal for intense attosecond radiation from an X-ray free-electron laser. Phys. Rev. Lett. 92, 224801 (10.1103/PhysRevLett.92.224801) 15245229

[32] Zholents AA 2005 Method of an enhanced self-amplified spontaneous emission for X-ray free electron lasers. Phys. Rev. Spec. Top.-Accel. Beams 8, 040701 (10.1103/PhysRevSTAB.8.040701)

[33] Tanaka T 2013 Proposal for a pulse-compression scheme in X-ray free-electron lasers to generate a multiterawatt, attosecond X-ray pulse. Phys. Rev. Lett. 110, 084801 (10.1103/PhysRevLett.110.084801) 23473154

[34] Shim CH, Parc YW, Kumar S, Ko IS, Kim DE 2018 Isolated terawatt attosecond hard X-ray pulse generated from single current spike. Sci. Rep. 8, 7463 (10.1038/s41598-018-25778-x) 29748612 PMC5945633

[35] Parc Y, Shim C, Kim D 2018 Toward the generation of an isolated TW-attosecond X-ray pulse in XFEL. Appl. Sci. 8, 1588 (10.3390/app8091588)

[36] Saldin EL, Schneidmiller EA, Yurkov MV 2006 Self-amplified spontaneous emission FEL with energy-chirped electron beam and its application for generation of attosecond X-ray pulses. Phys. Rev. Spec. Top.-Accel. Beams 9, 050702 (10.1103/PhysRevSTAB.9.050702)

[37] Baxevanis P, Duris J, Huang Z, Marinelli A 2018 Time-domain analysis of attosecond pulse generation in an X-ray free-electron laser. Phys. Rev. Accel. Beams 21, 110702 (10.1103/PhysRevAccelBeams.21.110702)

[38] Huang Z, Ding Y, Wu J 2010 *Three-dimensional analysis of frequency-chirped FELs*. Menlo Park, CA: SLAC National Accelerator Laboratory.

[39] Duris J *et al.* In press Superradiance in sinusoidally modulated electron beams. Phys. Rev. Accel. Beams.

[40] Ding Y, Huang Z, Ratner D, Bucksbaum P, Merdji H 2009 Generation of attosecond X-ray pulses with a multicycle two-color enhanced self-amplified spontaneous emission scheme. Phys. Rev. Spec. Top.-Accel. Beams 12, 060703 (10.1103/PhysRevSTAB.12.060703)

[41] MacArthur JP, Duris JP, Huang Z, Marinelli A, Zhang Z 2018 Self-modulation of a relativistic electron beam in a wiggler. In *Proc. 9th International Particle Accelerator Conf. (IPAC'18), Vancouver, BC, Canada, 29 April–4 May 2018*, number 9 in International Particle Accelerator Conference, pp. 4492–4495, Geneva, Switzerland. JACoW Publishing (10.18429/JACoW-IPAC2018-THPMK083)

[42] Marinelli A *et al.* 2015 High-intensity double-pulse X-ray free-electron laser. Nat. Commun. 6, 6369 (10.1038/ncomms7369) 25744344 PMC4366525

[43] Decker FJ, Bane KLF, Colocho W, Lutman AA, Sheppard JC 2017 Recent developments and plans for two bunch operation with up to 1 μs separation at LCLS. In Proc. of International Free Electron Laser Conference (FEL'17), Santa Fe, NM, USA, August 20–25 , pp. 288–291. Geneva, Switzerland: JACoW Publishing. (10.18429/JACoW-FEL2017-TUP023)

[44] Penco G *et al.* 2018 Two-bunch operation with ns temporal separation at the FERMI FEL facility. New J. Phys. 20, 053047 (10.1088/1367-2630/aac059)

[45] Lutman AA, Coffee R, Ding Y, Huang Z, Krzywinski J, Maxwell T, Messerschmidt M, Nuhn H-D 2013 Experimental demonstration of femtosecond two-color X-ray free-electron lasers. Phys. Rev. Lett. 110, 134801 (10.1103/PhysRevLett.110.134801) 23581326

[46] Marinelli A, Lutman AA, Wu J, Ding Y, Krzywinski J, Nuhn H-D, Feng Y, Coffee RN, Pellegrini C 2013 Multicolor operation and spectral control in a gain-modulated X-ray free-electron laser. Phys. Rev. Lett. 111, 134801 (10.1103/PhysRevLett.111.134801) 24116783

[47] Hara T *et al.* 2013 Two-colour hard X-ray free-electron laser with wide tunability. Nat. Commun. 4, 2919 (10.1038/ncomms3919) 24301682

[48] Lutman AA *et al.* 2016 Fresh-slice multicolour X-ray free-electron lasers. Nat. Photonics 10, 745–750. (10.1038/nphoton.2016.201)

[49] Guetg MW, Lutman AA, Ding Y, Maxwell TJ, Huang Z 2018 Dispersion-based fresh-slice scheme for free-electron lasers. Phys. Rev. Lett. 120, 264802 (10.1103/PhysRevLett.120.264802) 30004747

[50] Reiche S, Prat E 2016 Two-color operation of a free-electron laser with a tilted beam. J. Synchrotron Radiat. 23, 869–873. (10.1107/S1600577516007189) 27359134

[51] Chao Y-C, Qin W, Ding Y, Lutman AA, Maxwell T 2018 Control of the lasing slice by transverse mismatch in an X-ray free-electron laser. Phys. Rev. Lett. 121, 064802 (10.1103/PhysRevLett.121.064802) 30141681

[52] Ferrari E *et al.* 2016 Widely tunable two-colour seeded free-electron laser source for resonant-pump resonant-probe magnetic scattering. Nat. Commun. 7, 10343 (10.1038/ncomms10343) 26757813 PMC4735510

[53] Behrens GI *et al.* 2015 Femtosecond all-optical synchronization of an X-ray free-electron laser. Nat. Commun. 6, 5938 (10.1038/ncomms6938) 25600823 PMC4309427

[54] Beye M *et al.* 2012 X-ray pulse preserving single-shot optical cross-correlation method for improved experimental temporal resolution. Appl. Phys. Lett. 100, 121108 (10.1063/1.3695164)

[55] Schorb S *et al.* 2012 X-ray–optical cross-correlator for gas-phase experiments at the linac coherent light source free-electron laser. Appl. Phys. Lett. 100, 121107 (10.1063/1.3695163)

[56] Riedel R *et al.* 2013 Single-shot pulse duration monitor for extreme ultraviolet and X-ray free-electron lasers. Nat. Commun. 4, 1731 (10.1038/ncomms2754) 23591898

[57] Bionta MR *et al.* 2014 Spectral encoding method for measuring the relative arrival time between X-ray/optical pulses. Rev. Sci. Instrum. 85, 083116 (10.1063/1.4893657) 25173255

[58] Onn DG, Witek A, Qiu YZ, Anthony TR, Banholzer WF 1992 Some aspects of the thermal conductivity of isotopically enriched diamond single crystals. Phys. Rev. Lett. 68, 2806–2809. (10.1103/PhysRevLett.68.2806) 10045497

[59] Lindsay L, Broido DA, Reinecke TL 2013 First-principles determination of ultrahigh thermal conductivity of boron arsenide: a competitor for diamond? Phys. Rev. Lett. 111, 025901 (10.1103/PhysRevLett.111.025901) 23889420

[60] Trigo M *et al.* 2013 Fourier-transform inelastic X-ray scattering from time- and momentum-dependent phonon-phonon correlations. Nat. Phys. 9, 790–794. (10.1038/nphys2788)

[61] Harmand M *et al.* 2013 Achieving few-femtosecond time-sorting at hard X-ray free electron lasers. Nat. Phot. 7, 215–218. (10.1038/nphoton.2013.11)

[62] Hartmann N *et al.* 2014 Sub-femtosecond precision measurement of relative X-ray arrival time for free-electron lasers. Nat. Photon. 8, 706–709. (10.1038/nphoton.2014.164)

[63] Olivier C *et al.* 2017 Accurate prediction of X-ray pulse properties from a free-electron laser using machine learning. Nat. Comm. 8, 15461 (10.1038/ncomms15461) PMC5465316 28580940

[64] Frühling U 2011 Light-field streaking for FELs. J. Phys. B: At. Mol. Opt. Phys. 44, 243001 (10.1088/0953-4075/44/24/243001)

[65] Düsterer S *et al.* 2011 Femtosecond X-ray pulse length characterization at the Linac Coherent Light Source free-electron laser. New J. Phys. 13, 093024 (10.1088/1367-2630/13/9/093024)

[66] Grguras I *et al.* 2012 Ultrafast X-ray pulse characterization at free-electron lasers. Nat. Photonics 6, 852–857. (10.1038/nphoton.2012.276)

[67] Helml W *et al.* 2014 Measuring the temporal structure of few-femtosecond free-electron laser X-ray pulses directly in the time domain. Nat. Photonics 8, 950–957. (10.1038/nphoton.2014.278)

[68] Juranić PN *et al.* 2014 High-precision X-ray FEL pulse arrival time measurements at SACLA by a THz streak camera with Xe clusters. Opt. Express 22, 30 004–30 012. (10.1364/OE.22.030004) 25606930

[69] Hoffmann MC *et al.* 2018 Femtosecond profiling of shaped X-ray pulses. New J. Phys. 20, 033008 (10.1088/1367-2630/aab548)

[70] Helml W *et al.* 2017 Ultrashort free-electron laser X-ray pulses. Appl. Sci. 7, 915 (10.3390/app7090915)

[71] Chini M, Zhao K, Chang Z 2014 The generation, characterization and applications of broadband isolated attosecond pulses. Nat. Photonics 8, 178–186. (10.1038/nphoton.2013.362)

[72] Constant E, Taranukhin VD, Stolow A, Corkum PB 1997 Methods for the measurement of the duration of high-harmonic pulses. Phys. Rev. A 56, 3870–3878. (10.1103/PhysRevA.56.3870)

[73] Zhao ZX, Chang Z, Tong XM, Lin CD 2005 Circularly-polarized laser-assisted photoionization spectra of argon for attosecond pulse measurements. Opt. Express 13, 1966–1977. (10.1364/OPEX.13.001966) 19495079

[74] Eckle P, Pfeiffer AN, Cirelli C, Staudte A, Dörner R, Muller HG, Büttiker M, Keller U 2008 Attosecond ionization and tunneling delay time measurements in helium. Science 322, 1525–1529. (10.1126/science.1163439) 19056981

[75] Hartmann N *et al.* 2018 Attosecond time-energy structure of X-ray free-electron laser pulses. Nat. Photonics 12, 215–220. (10.1038/s41566-018-0107-6)

[76] Kazansky AK, Bozhevolnov AV, Sazhina IP, Kabachnik NM 2016 Interference effects in angular streaking with a rotating terahertz field. Phys. Rev. A 93, 013407 (10.1103/PhysRevA.93.013407)

[77] Kazansky AK, Sazhina IP, Nosik VL, Kabachnik NM 2017 Angular streaking and sideband formation in rotating terahertz and far-infrared fields. J. Phys. B: At. Mol. Opt. Phys. 50, 105601 (10.1088/1361-6455/aa69e9)

[78] Spanner M, Smirnova O, Corkum PB, Ivanov MY 2004 Reading diffraction images in strong field ionization of diatomic molecules. J. Phys. B: At. Mol. Opt. Phys. 37, L243–L250. (10.1088/0953-4075/37/12/L02)

[79] Li S *et al.* 2018 A co-axial velocity map imaging spectrometer for electrons. AIP Adv. 8, 115308 (10.1063/1.5046192)

[80] Kitzler M, Milosevic N, Scrinzi A, Krausz F, Brabec T 2002 Quantum theory of attosecond XUV pulse measurement by laser dressed photoionization. Phys. Rev. Lett. 88, 173904 (10.1103/PhysRevLett.88.173904) 12005757

[81] Li S *et al.* 2018 Characterizing isolated attosecond pulses with angular streaking. Opt. Express 26, 4531–4547. (10.1364/OE.26.004531) 29475303

[82] Fechner S, Dimler F, Brixner T, Gerber G, Tannor DJ 2007 The von Neumann picture: a new representation for ultrashort laser pulses. Opt. Express 15, 15 387–15 401. (10.1364/OE.15.015387) 19550824

[83] Drescher M *et al.* 2002 Time-resolved atomic inner-shell spectroscopy. Nature 419, 803–807. (10.1038/nature01143) 12397349

[84] Verhoef AJ, Mitrofanov AV, Nguyen XT, Krikunova M, Fritzsche S, Kabachnik NM, Drescher M, Baltuška A 2011 Time-and-energy-resolved measurement of Auger cascades following Kr 3d excitation by attosecond pulses. New J. Phys. 13, 113003 (10.1088/1367-2630/13/11/113003)

[85] Verhoef AJ, Mitrofanov AV, Nguyen XT, Krikunova M, Fritzsche S, Kabachnik NM, Drescher M, Baltuška A 2011 Time-and-energy resolved measurement of the cascaded Auger decay in krypton. Laser Phys. 21, 1270–1274. (10.1134/S1054660X11130263)

[86] Kazansky AK, Sazhina IP, Kabachnik NM 2019 Angular streaking of Auger-electrons by THz field. J. Phys. B: At. Mol. Opt. Phys. 52, 045601 (10.1088/1361-6455/aafa33)

[87] McFarland BK *et al.* 2014 Ultrafast X-ray Auger probing of photoexcited molecular dynamics. Nat. Commun. 5, 4235 (10.1038/ncomms5235) 24953740

